# CD24 blunts the sensitivity of retinoblastoma to vincristine by modulating autophagy

**DOI:** 10.1002/1878-0261.12708

**Published:** 2020-06-13

**Authors:** Jie Sun, Dongju Feng, Huiyu Xi, Jiajing Luo, Zewei Zhou, Qinghuai Liu, Yun Chen, Qing Shao

**Affiliations:** ^1^ Department of Ophthalmology the First Affiliated Hospital of Nanjing Medical University Nanjing China; ^2^ Department of Immunology Key Laboratory of Immune Microenvironment and Disease Nanjing Medical University Nanjing China; ^3^ Department of Ophthalmology Xuzhou First People's Hospital of Xuzhou Medical University Xuzhou Eye Research Institute Xuzhou China; ^4^ Jiangsu Key Lab of Cancer Biomarkers, Prevention and Treatment Collaborative Innovation Center for Cancer Personalized Medicine Nanjing Medical University Nanjing China

**Keywords:** autophagy, CD24, lipid raft, retinoblastoma, vincristine

## Abstract

Retinoblastoma (RB) is the most common childhood malignant intraocular tumor. The clinical efficacy of vincristine (VCR) in the treatment of RB is severely limited by drug resistance. Here, we found that CD24, a GPI‐anchored protein, was overexpressed in human RB tissues and RB cell lines, and was associated with the sensitivity of RB cells in response to VCR therapy. We demonstrated that CD24 plays a critical role in impairing RB sensitivity to VCR via regulating autophagy. Mechanistically, CD24 recruits PTEN to the lipid raft domain and regulates the PTEN/AKT/mTORC1 pathway to activate autophagy. Lipid raft localization was essential for CD24 recruitment function. Collectively, our findings revealed a novel role of CD24 in regulating RB sensitivity to VCR and showed that CD24 is a potential target for improving chemotherapeutic sensitivity and RB patient outcomes.

## Graphical abstract

Chemotherapy failure is the major cause of recurrence and poor prognosis in retinoblastoma (RB) patients. We found that CD24 is overexpressed in human RB tissues and RB cell lines, and is associated with the sensitivity of RB cells to VCR therapy. CD24, a GPI‐anchored protein linked to membrane lipid raft microdomains, recruits PTEN to the lipid raft domain and regulates the PTEN/AKT/mTORC1 pathway to activate autophagy. Our study shows that CD24 blunts the sensitivity of RB to VCR via activating autophagy.

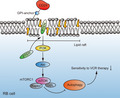

**Abbreviations**
ABC: ATP‐binding cassette
CQ: chloroquine
CRC: colorectal cancer
DEGs: differentially expressed genes
FACS: flow cytometry
GPI: glycosylphosphatidylinositol
HCC: hepatocellular carcinoma
IHC: immunohistochemistry
PIPLC: phosphatidylinositol‐specific phospholipase C
PLL: poly‐l‐lysine
RB: retinoblastoma
siRNAs: short‐interfering RNAs
TNBC: triple‐negative breast cancer
VCR: vincristine

## 1 Introduction

Retinoblastoma (RB), the most common primary intraocular cancer of childhood, is a malignancy arising in the developing retina. It represents 4% of all pediatric malignancies (Bai *et al*., 2016; Shields and Shields, 2004). The treatment of RB is multimodal. Basic treatment methods are chemotherapy, radiation therapy, enucleation, and focal treatment including transpupillary thermotherapy, cryotherapy, and laser photocoagulation, all playing a vital role (Yanik *et al*., 2015). Recently, chemotherapy has become the most common eye‐sparing approach. The standard chemotherapeutic agents used for intraocular RB are the combination of vincristine, etoposide, and carboplatin (Shukla *et al*., 2017). However, drug resistance of tumor cells easily leads to chemotherapy failure (Jiang and Zhang, 2016). Resistance to chemotherapy leading to poor outcome and survival remains a challenge for developing strategies for therapeutic interventions. Thus, it is of paramount importance to elucidate the mechanisms of RB chemotherapy resistance.

Accumulating evidence indicates that an increasing number of proteins involved in the development of several malignant cancers are being detected to be associated with lipid rafts (Staubach and Hanisch, 2011). It is known that lipid rafts function as platforms for the recruitment of signaling proteins to favor protein–protein interactions and facilitate activation of signal transduction cascades as well as receptor‐mediated signaling (Mollinedo and Gajate, 2015). CD24 is a highly glycosylated protein that is linked to membrane lipid raft microdomains via a glycosylphosphatidylinositol (GPI) anchor. Recent studies have shown that CD24 correlates with poor prognosis in many types of tumor tissues, including gliomas (Deng *et al*., 2012), hepatocellular carcinoma (HCC) (Wan *et al*., 2016), and breast cancer (Jing *et al*., 2018). Furthermore, CD24 may play a role in chemotherapy resistance in triple‐negative breast cancer (TNBC) (Deng *et al*., 2017), HCC (Lu *et al*., 2018), and endometrial cancer (Ono *et al*., 2015), further implicating it as a potential drug target (Ono *et al*., 2015; Runz *et al*., 2008; Schabath *et al*., 2006). Studies have suggested that CD24 regulates different signaling pathways in various cancer cells (Eyvazi *et al*., 2018). Lipid raft localization is essential for the signal transduction capacity of CD24 (Runz *et al*., 2008). It is reported that the GPI‐linked surface molecule CD24 complexes with members of the c‐Src family and then activates them in a small‐cell lung cancer (Zarn *et al*., 1996). Active form of Src activates STAT3 molecules and then induces cancer‐promoting activities involved in cell proliferation, metastasis, or resistance to apoptosis (Bretz *et al*., 2012).

Macroautophagy (referred to as autophagy hereafter) is an evolutionarily conserved and highly regulated homeostatic process involving the formation of double‐membraned vesicles called autophagosomes that engulf cellular proteins and organelles for delivery to the lysosome (Levy *et al*., 2017). Accumulating evidence suggests that autophagy plays significant roles in the ability of cancers to develop resistance to chemotherapy (Wu *et al*., 2014; Yu *et al*., 2017). Autophagy is activated as a protective mechanism to mediate the acquired resistance phenotype of tumor cells under chemotherapy, and inhibition of autophagy can resensitize previously resistant cancer cells and increase the cytotoxicity of chemotherapeutic drugs (Sui *et al*., 2013). Autophagy is regulated by various signaling pathways in which the PI3K‐Akt‐mTOR signaling pathway plays a central role in the regulation of autophagy. Blockade of autophagy through the PI3K‐Akt‐mTOR pathway powerfully overcomes chemotherapy resistance and resensitizes the tumor cells to anticancer therapy (Kumar *et al*., 2015).

In RB, CD24 expression is an important marker for prediction of the severity of RB and the prognosis of the patients (Li *et al*., 2012). In our study, we found that CD24 is positively correlated with sensitivity to VCR therapy. However, the cellular mechanisms involved in activity of CD24 in RB remain unclear. In this report, we investigated the roles of CD24 in regulating the sensitivity of RB to VCR. We found that inhibition of CD24 could downregulate the activation of autophagy through the PTEN/Akt/mTORC1 pathway, resulting in increased susceptibility to VCR. Our findings indicated that CD24 plays a key role in regulating the response of RB cells to VCR therapy through induction of autophagy. It provides a new therapeutic target, which facilitates chemotherapy of RB.

## 2 Materials and methods

### 2.1 Antibodies and reagents

The information on all antibodies and reagents is listed in Table S1.

### 2.2 Cell culture

The human retinoblastoma cell lines WERI‐Rb‐1 (HTB‐169) and Y79 (HTB‐18), and the normal human retinal pigment epithelial cell line ARPE‐19 (CRL‐2302) were purchased from the American Type Culture Collection (ATCC, Manassas, VA, USA) and authenticated by short tandem repeat DNA profiling analysis. All the cancer cells were cultured in RPMI‐1640 medium (Gibco, Grand Island, NY, USA) containing 1% penicillin–streptomycin (Gibco) and 10% heat‐inactivated FBS (Invitrogen, Carlsbad, CA, USA). The normal human retinal pigment epithelial cell line ARPE‐19 was grown in DMEM/F12 HEPES medium (Gibco) with 1% penicillin–streptomycin and 10% FBS. All cells were cultured in a humidified incubator with 5% CO_2_ at 37 °C.

### 2.3 Tissue microarray

Retinoblastoma and normal eye tissue microarrays were obtained from Alenabio (Xian, China) for immunohistochemistry (IHC). This tissue microarray is comprised of 14 primary RB tumor samples and 6 normal eye tissue samples. Relevant clinical pathologic features were all obtained from the patients' files (Table S2). Pathology diagnosis with RB was confirmed by an attending pathologist. The clinical staging was performed according to the tumor‐node‐metastasis classification system.

### 2.4 Immunohistochemistry

Tissue samples were fixed in 10% formalin and embedded in paraffin. For immunohistochemical staining, slides were deparaffinized in xylene, hydrated in series of alcohol, treated with an antigen retrieval solution (10 mm sodium citrate buffer; pH 6.0), and incubated with 1.5% H_2_O_2_ for 15 min at room temperature, to quench the endogenous peroxidase activity. After blocking, tissue sections were incubated with primary antibodies overnight at temperature. Immunodetection was performed using DAB Peroxidase Substrate Kit (VECTOR, Burlingame, CA, USA). The sections were then counterstained with hematoxylin. All staining was evaluated by three independent pathologists who were blinded to the clinical details. The staining scores were determined based on the percentage of positive cells and the staining intensity (IHC score = staining percentage × intensity).

### 2.5 Western blot analysis

Cells were lysed in RIPA lysis buffer with a protease and phosphatase inhibitor cocktail (Thermo Fisher Scientific, Waltham, MA, USA). The protein concentration was measured using BCA Protein Assay Kit (Thermo Fisher Scientific). Proteins in lysates (30–50 μg total protein) were separated by SDS/PAGE and transferred onto the PVDF membrane (Millipore, Billerica, MA, USA). The membrane was blocked in 5% nonfat dry milk for 1 h and then incubated with primary antibodies at 4 °C overnight. Secondary antibodies conjugated to HRP were used, and immunoreactive bands were developed with the ECL western blotting system. β‐actin and α‐tubulin were used as a control for whole‐cell lysates.

### 2.6 Flow cytometry

Cells were harvested and suspended in ice‐cold PBS with 1% FBS. Cells were stained by fluorescently conjugated antibodies and incubated at 4 °C for 30 min in the dark. Samples were acquired on a CytoFLEX (Beckman Coulter, Shanghai, China), and data were analyzed with flowjo software (Treestar, San Carlos, CA, USA).

### 2.7 Quantitative real‐time PCR

Total RNAs of cells was extracted with TRIzol reagent (Invitrogen). For cDNA synthesis, total RNA was reverse‐transcribed using the PrimeScript RT Reagent Kit (Takara, Beijing, China). Quantitative real‐time PCR was performed in triplicates using PowerUp SYBR Green Master Mix (Thermo Fisher Scientific) on an Applied Biosystems StepOnePlus Real‐Time PCR machine (Thermo Fisher Scientific). Relative quantification of target gene expression was determined using the comparative *C*_t_ method. β‐actin was used as the internal control, and all primers used are listed in Table S3.

### 2.8 Cell survival and apoptosis assays

Cell survival was measured using Cell Counting Kit‐8 (Bimake, Shanghai, China) assays. Cells were plated in 96‐well plates at a density of 1.0 × 10^4^ cells per well with 100 μL culture medium and treated with different concentrations of VCR (0–5.12 μm) for 48 h. Then, 10 μL of CCK‐8 solution was added to the cells, which were then incubated for 2 h at 37 °C. Absorbance was measured at 450 nm.

Apoptosis was determined by western blot analysis (detecting the cleaved level of apoptosis‐related proteins: cleaved caspase‐3 and cleaved PARP) and flow cytometry with the FITC Annexin V Apoptosis Detection Kit (BD Pharmingen, San Diego, CA, USA) (Zhou *et al*., 2018). FITC Annexin V and PI staining can divide cells into three categories. Cells that are considered viable are FITC Annexin V‐ and PI‐negative; cells that are in early apoptosis are FITC Annexin V‐positive and PI‐negative, and cells that are in late apoptosis or already dead are both FITC Annexin V‐ and PI‐positive. The apoptosis rate is measured by the percentage of early and late apoptotic cells.

### 2.9 RNA sequencing and data analysis

Total RNA of each sample was extracted using RNeasy Mini Kit (Qiagen, Hilden, Germany) and qualified by Agilent 2100 Bioanalyzer (Agilent Technologies, Palo Alto, CA, USA). Next‐generation sequencing library preparations were constructed according to the manufacturer's protocol (NEBNext Ultra RNA Library Prep Kit for Illumina). Samples were run on an Illumina HiSeq instrument (Illumina, San Diego, CA, USA) for 2 × 150‐bp paired‐end sequencing. The sequences were processed and analyzed by GENEWIZ (Nanjing, China).

For data analysis, we used hisat2 (v2.0.1, Johns Hopkins University, Baltimore, MD, USA) to map the cleaned data to reference genome. Differential expression analysis used the DESeq Bioconductor package. *P*‐value of genes was set < 0.05 to detect differential expressed ones. The signal pathways were analyzed by the primary public database Kyoto Encyclopedia of Genes and Genomes (KEGG) (https://www.kegg.jp) and enriched by the tool Pathway Maps. Gene set enrichment analysis was performed using gsea software version 3.0 with Molecular Signature Database (MSigDB) (http://software.broadinstitute.org/gsea/index.jsp).

### 2.10 Transfection and transduction

Short‐interfering RNAs (siRNAs) and the CD24 overexpression plasmid were designed and synthesized by Shanghai Genepharma Co., Ltd (Shanghai, China) and used for transfection. Transfection was performed using Lipofectamine 3000 (Invitrogen) following the manufacturer's instructions. Prior to transfection, six‐well plates were coated with poly‐l‐lysine (PLL) to make the RB suspension cells adhere to the bottom of each plate (Beta *et al*., 2014). Briefly, 5 × 10^5^ cells/well were plated in PLL‐coated 6‐well plates. siRNA transfection was carried out when RB cells were cultured to 50–60% confluence. Lentiviruses for human CD24 knockdown and RFP‐GFP‐LC3 lentiviruses were from Shanghai GeneChem Co., Ltd. (Shanghai, China). FFluc‐GFP‐Puro lentiviruses were purchased from Shanghai Realgene Bio‐tech, Inc. (Shanghai, China). For transduction, RB cells were plated at 0.5 × 10^5^ cells·mL^−1^ in 24‐well plates and transduced with lentiviral particles (multiplicity of infection of 20) with 5 µg·mL^−1^ polybrene. Sequences of siRNAs and shRNAs are listed in Table S4.

### 2.11 Immunofluorescence and confocal microscopy

For immunofluorescent staining, cells were plated in PLL‐coated glass‐bottom cell culture dishes. The cells were fixed with 4% paraformaldehyde for 30 min and blocked with 1% BSA for 1 h at room temperature. Subsequently, cells were incubated with indicated primary antibodies overnight at 4 °C. Cells were then incubated with secondary antibodies coupled to Alexa Fluor dyes at room temperature for 1 h. Nuclei were counterstained with DAPI. Cells were observed using a LSM710 confocal microscope (ZEISS, Jena, Germany). To perform image‐based analysis for autophagy, RB cells with stable expression of RFP‐GFP‐LC3 were obtained by viral infection and puromycin screening. The principle of the assay is based on different pH stability of GFP and RFP fluorescent proteins. Autophagosomes are visualized as yellow puncta, while autolysosomes are manifest as red puncta. LC3 puncta were examined with a confocal fluorescence microscope.

### 2.12 Electron microscopy

Cells were immobilized at room temperature with 2.5% glutaraldehyde with 0.1 m sodium cacodylate for 2 h and postfixed with 1% osmium tetroxide for 1 h. Then, the cells were dehydrated, embedded, and sectioned. Images were acquired using a Tecnai G2 Spirit BioTWIN transmission electron microscope (FEI, Hillsboro, OR, USA) after the samples were stained with 3% uranyl acetate and lead citrate.

### 2.13 Isolation of lipid raft

Cells (1 × 10^8^) were washed twice with PBS at 4 °C and then dissolved in 2 mL MES buffer (25 mm MES, pH 6.5, 150 mm NaCl) containing 1% Triton X‐100. The extract was adjusted to 40% sucrose by adding 2 mL of 80% sucrose, placed at the bottom of an ultracentrifuge tube, and overlaid with 1 mL of 35%, 30%, 25%, 20%, 15%, and 5% sucrose in MES buffer as above but minus the Triton X‐100. The mixture was centrifuged for 18 h at 4 °C in a Beckman SW50.1 rotor (Beckman Coulter, Inc., Indianapolis, IN, USA) at 200 000 ***g***. Ten 1 mL fractions were collected from the top to the bottom of the tubes. An equal volume of MES buffer was added to these fractions. The complexes were collected by centrifugation for 15 min at 3000 ***g***. Pellets were resuspended in Laemmli buffer, boiled, separated by SDS/PAGE, and analyzed by western blotting.

### 2.14 Orthotopic RB mouse model

Six‐week‐old BALB/c nude male mice were used to construct orthotopic RB mouse model. Mice were anesthetized using intraperitoneal injections of 4.3% chloral hydrate and topical application of 0.4% oxybuprocaine. The pupil was dilated with 1–2 drops of tropicamide/phenylephrine eye drops. Cells (2 × 10^5^) labeled with luciferase prepared in 2 µL PBS were injected slowly into the vitreous of the left eye with a Hamilton syringe under the microscope. VCR and chloroquine (CQ) were administered by intraperitoneal injection, twice per week for three weeks. Bioluminescent images were acquired by an IVIS Spectrum imaging system (PerkinElmer, Waltham, MA, USA). Bioluminescence signal was quantified using the living image software (PerkinElmer). All experiments were performed in accordance with a protocol approved by the Institutional Animal Care and Use Committee of Nanjing Medical University.

### 2.15 Statistical analysis

All statistical analyses were performed using spss 13.0 software (SPSS Inc., Chicago, IL, USA) or graphpad prism 6.0 (GraphPad Software, Inc., San Diego, CA, USA). Data are expressed as means ± standard deviation (SD). Unpaired numerical data were compared using an unpaired *t‐*test (two groups) or analysis of variance (ANOVA; more than two groups). All statistical analyses were performed using two‐tailed *P*‐values, and *P*‐values less than 0.05 were considered to indicate statistical significance. The quantitative data were analyzed using Student's *t*‐test.

## 3 Results

### 3.1 CD24 is overexpressed in human RB tissues and RB cell lines

**Fig. 1:**
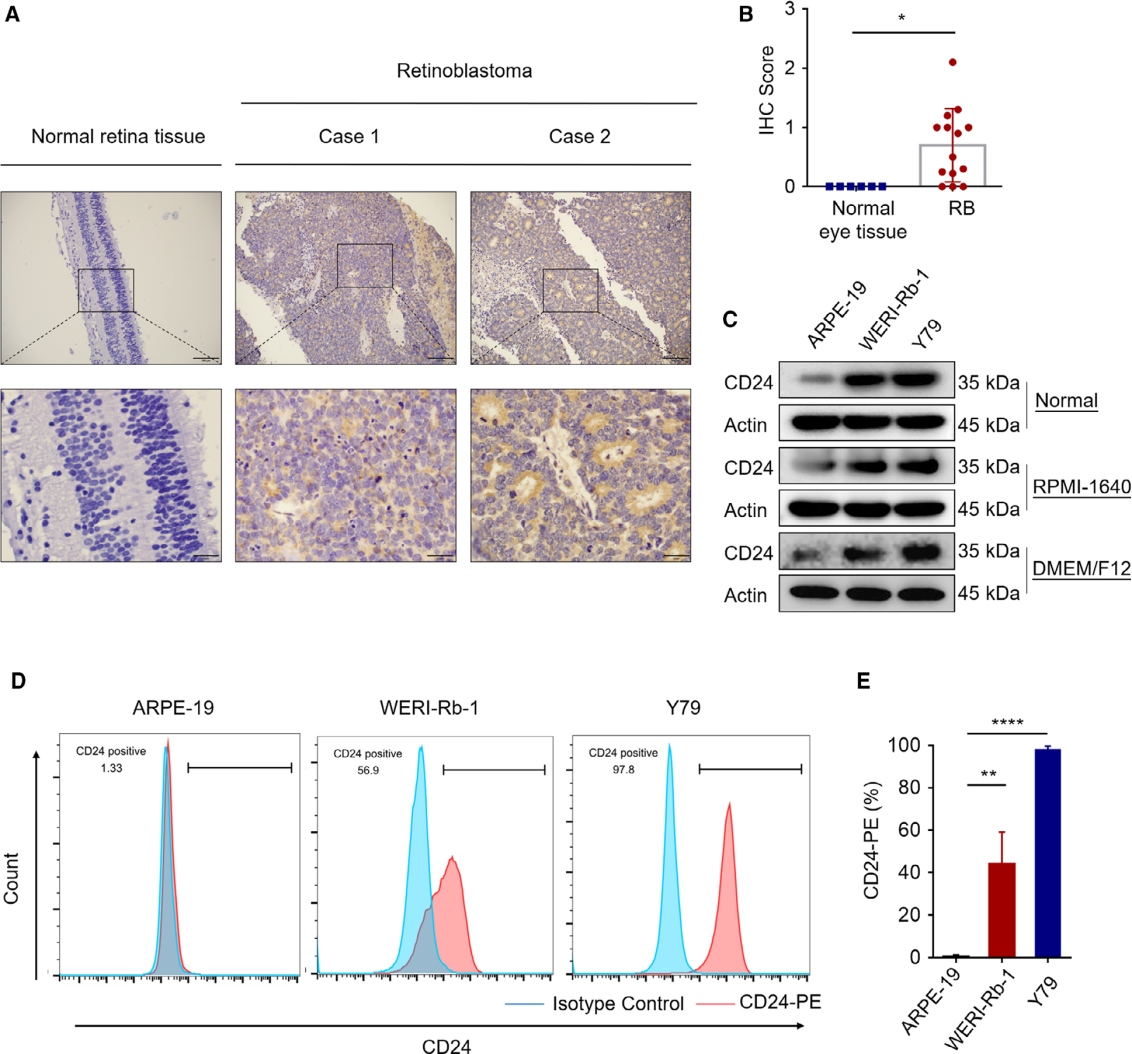
CD24 expression in human RB tissues and cell lines. (A) Representative images of IHC analysis of CD24 protein expression in RB tumor tissues and normal eye tissues. Scale bars, 100 μm (up). Scale bars, 25 μm (down). (B) Quantitative analysis of CD24 protein expression based on staining index in RB (*n* = 14) and normal eye tissues (*n* = 6, Student's *t*‐test). (C) Western blot of CD24 expression in ARPE‐19, WERI‐Rb‐1, and Y79 cells cultured in RPMI‐1640 medium or DMEM/F12 HEPES medium. (D, E) Flow cytometry analysis of CD24‐PE staining in ARPE‐19, WERI‐Rb‐1, and Y79 cells. Representative plots (left) and quantification (right) are shown (*n* = 3, Student's *t*‐test). Quantitative data are presented as mean ± SD (*P < 0.05, **P < 0.01, ****P < 0.0001).

Previous studies have shown that CD24 is associated with poor prognosis in RB (Ishaq *et al*., 2016; Li *et al*., 2012). To investigate whether CD24 is overexpressed in patient RB tissues, we examined its expression by IHC in 14 primary RB tumor samples and 6 normal eye tissue samples. In comparison with normal eye tissues, the expression level of CD24 was identified to be greater in the RB tissues (Fig. 1A,B). Next, we asked whether CD24 is expressed at high levels in RB cell lines. We examined the protein expression levels of CD24 by western blotting and flow cytometry (FACS) analysis in a panel of retinal cell lines, including human retinal pigment epithelial cell line ARPE‐19 and RB cell lines WERI‐Rb‐1 and Y79. CD24 overexpression was observed in the WERI‐Rb‐1 and Y79 cell lines with *in vivo* tumor‐forming ability. In contrast, the immortalized nontumorigenic cell line ARPE‐19, which is incapable of tumor formation *in vivo*, exhibited extremely low CD24 expression (Fig. 1C–E). According to the recommendation of the American Type Culture Collection (ATCC), ARPE‐19 cell line was cultured in DMEM/F12 HEPES medium and RB cell lines were grown in RPMI‐1640 medium. In order to exclude the effect of culture conditions on the protein expression level of CD24, we detected the protein expression level of CD24 in ARPE‐19 and RB cell lines cultured in the same media (RPMI‐1640 medium or DMEM/F12 HEPES medium). Regardless of whether these three cell lines are cultured in RPMI‐1640 or DMEM/F12 HEPES medium, CD24 is highly expressed in RB cell lines compared with ARPE‐19 cell line (Fig. 1C). These results indicated that CD24 is overexpressed in RB and might play a role in tumor development *in vivo*.

### 3.2 CD24 impedes the sensitivity of RB cells to VCR

**Fig. 2:**
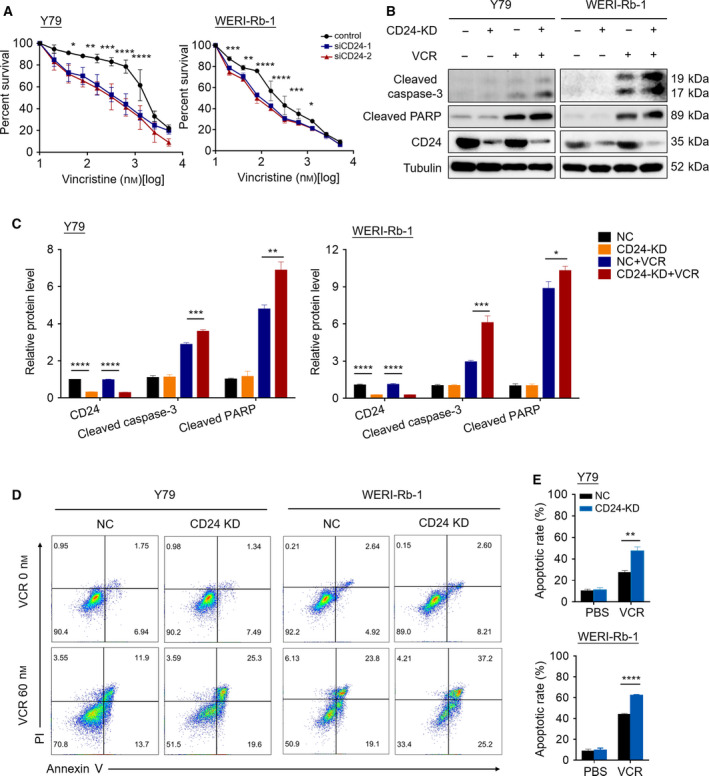
CD24 impedes the sensitivity of RB cells to VCR. (A) The CCK‐8 assay was performed in CD24‐silenced RB cell lines and their nontarget control cells after exposure to a serial dose–response of VCR for 48 h (*n* = 3, Student's *t*‐test). (B) Western blotting was performed in CD24 knockdown (CD24 KD) and control RB cells treated with PBS or VCR (60 nm) for 48 h. (C) Quantification analysis of the western blot image shown in (B) using imagej software (National Institutes of Health, Bethesda, MD, USA). (D, E) Fluorescence‐activated cell sorting (FACS) showing the apoptotic rate of cells stained with Annexin V‐APC/PI following 48 h of treatment with PBS or VCR. Representative plots (D) and quantification (E) are shown (*n* = 3, Student's *t‐*test). Quantitative data are presented as mean ± SD (*P < 0.05, **P < 0.01, ***P < 0.001, ****P < 0.0001).

To investigate whether CD24 expression affects the sensitivity of RB cells to VCR, we knocked down *CD24* mRNA and protein expression in RB cells via two human *CD24*‐targeted RNA interference expression vectors (siRNA). Knockdown efficiency was confirmed by qPCR and western blotting (Fig. S1A,B). Next, we performed VCR sensitivity assays in the Y79 and WERI‐Rb‐1 cell lines with CD24 knockdown. We found that CD24 knockdown RB cell lines had significantly higher sensitivity to VCR compared with nontargeted control cells (Fig. 2A). As shown in Fig. S1A,B, CD24‐siRNA2 (siCD24‐2) displayed maximal knockdown efficiency. Therefore, we added a hairpin structure to siCD24‐2 and established stable CD24 knockdown (CD24 KD) RB cells. CD24 KD RB cells were used for follow‐up experiments. We further evaluated the effects of VCR on induction of cell apoptosis by measuring activated caspases using a western blot assay. Western blotting showed that VCR induced the cleavage of caspase‐3 and PARP, and these effects were enhanced by CD24 knockdown in Y79 and WERI‐Rb‐1 cells, indicating increased susceptibility to VCR‐induced cell death (Fig. 2B,C). Consistent with this, FACS measurements of Annexin V/PI staining (Fig. 2D,E) revealed that the apoptosis rate of CD24 KD RB group cells increased by approximately 20.07 ± 1.97% in Y79 cells and 18.53 ± 0.95% in WERI‐Rb‐1 cells relative to control group cells challenged with VCR. These findings demonstrated that CD24 blunts the sensitivity of RB to VCR.

### 3.3 CD24 regulates autophagy activation in RB cells under VCR challenge

**Fig. 3:**
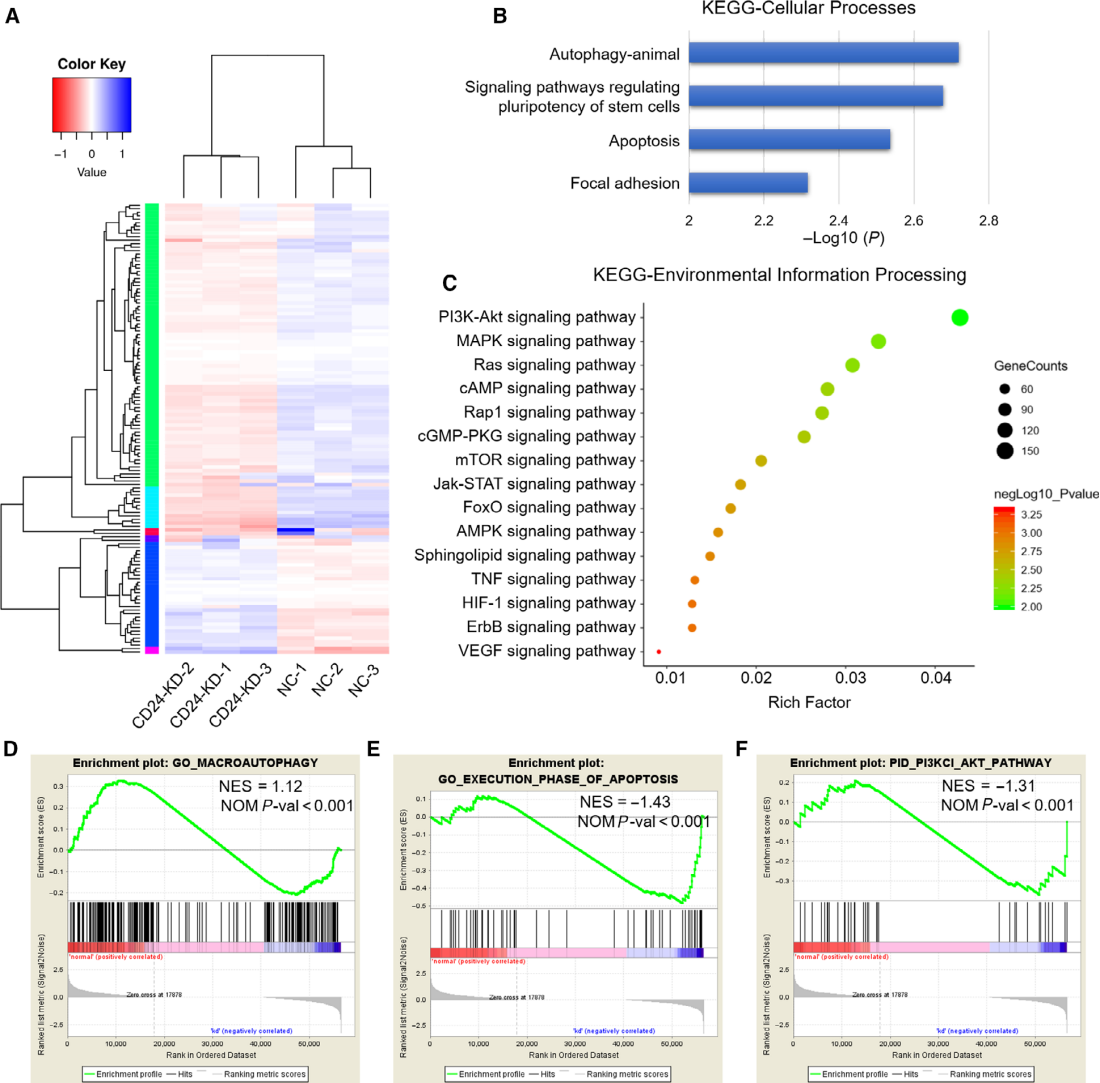
Comparative transcriptome analysis of CD24 KD and control RB cells. (A) Heatmap representation of the significantly DEGs from RNA‐seq data after treatment with VCR (60 nm) for 48 h. (B) KEGG pathway significant enrichment analysis for DEGs in the Cellular Processes category. (C) KEGG pathway significant enrichment analysis for DEGs in the Environmental Information Processing category. The horizontal axis indicates rich factor, and vertical ordinates are the terms of the KEGG pathways. The bubble size shows the number of DEGs, and the color of the bubble represents −log_10_(*P* value): Logarithmic conversion of Fisher's exact test *P* value. (D) GSEA of the ‘GO MACROAUTOPHAGY’ gene set. (E) GSEA of the ‘GO EXECUTION PHASE OF APOPTOSIS’ gene set. (F) GSEA of the ‘PID PI3KCI AKT PATHWAY’ gene set.

To interrogate the mechanism by which CD24 regulates the response of RB cells to VCR, we performed an RNA‐seq analysis and compared the gene expression profiles between CD24 KD and control Y79 cells after exposure to VCR (60 nm) for 48 h (Fig. 3A). Impressively, the KEGG pathway enrichment analysis showed that autophagy was the most significant pathway in the Cellular Processes category (Fig. 3B). Moreover, GSEA revealed that the gene set of ‘GO MACROAUTOPHAGY’ was significantly enriched in control Y79 cells and ‘GO EXECUTION PHASE OF APOPTOSIS’ was significantly enriched in CD24‐KD Y79 Cells (Fig. 3D,E). Consistent with these observations, the expression of multiple autophagy signaling elements including Beclin 1, Atg3, Atg5, Atg12, and Atg16L1 was increased in control RB cells (Fig. S2A,B).

**Fig. 4:**
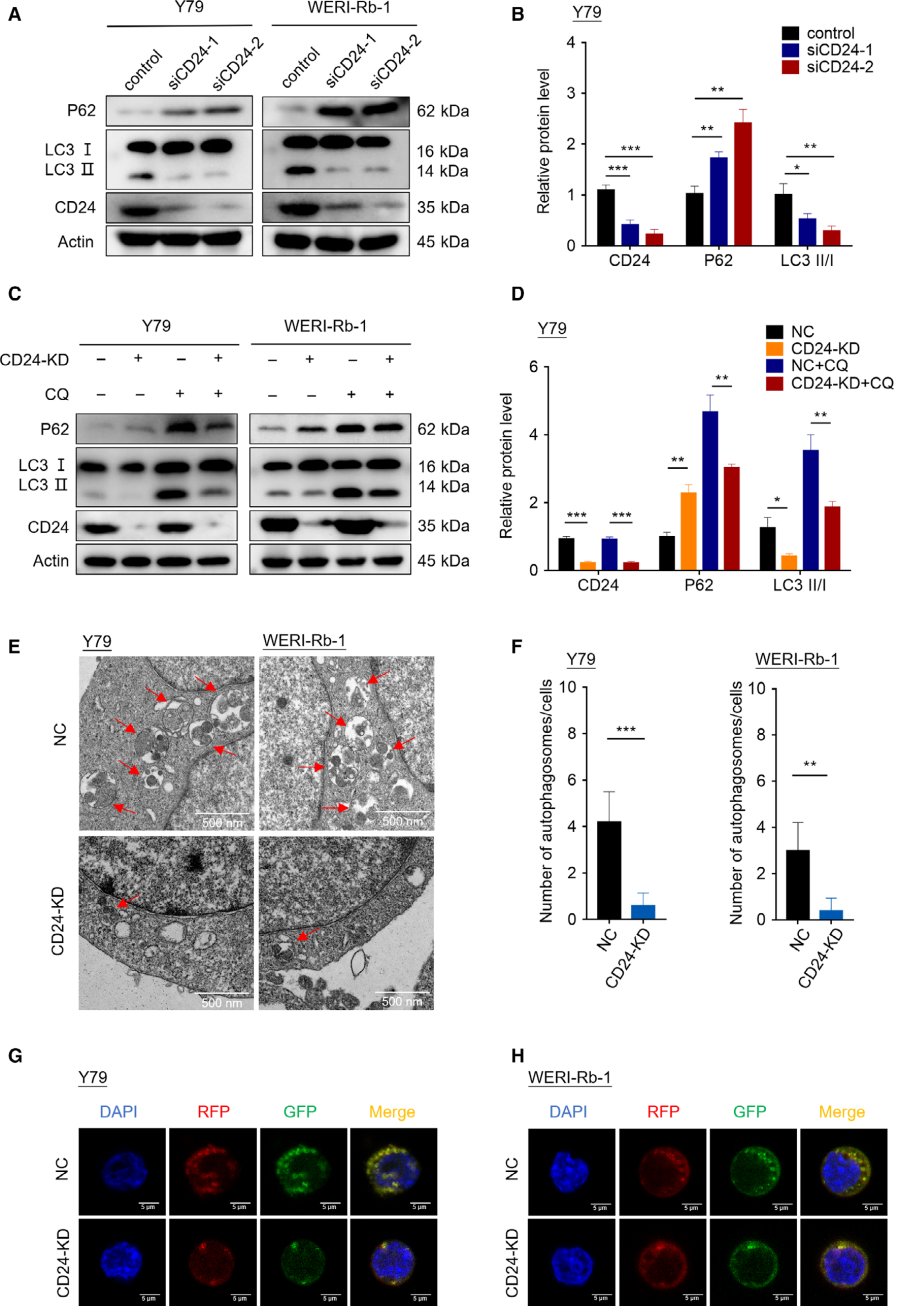
CD24 regulates autophagy activation in RB cells under VCR challenge. (A) The expression of LC3B and p62 protein was analyzed by western blotting. (B) The western blot images of Y79 cells in (A) were quantitatively analyzed using imagej software (National Institutes of Health, Bethesda, MD, USA). (C) Western blotting was performed in RB cells with or without CQ (20 μm) treatment for 1 h. (D) The western blot images of Y79 cells in (C) were quantitatively analyzed using imagej software. (E) Autophagosomes were observed by transmission electron microscopy in CD24 KD and control RB cells treated with CQ for 1 h. Scale bars, 0.5 μm. (F) Statistical analysis was performed to calculate the number of autophagosomes in RB cells shown by transmission electron microscopy (*n* = 5, Student's *t*‐test). (G, H) Y79 cells (G) and WERI‐Rb‐1 cells (H) that stably expressed RFP‐GFP‐LC3 fusion protein were treated with CQ for 1 h. The fluorescence signals were visualized by confocal immunofluorescence microscopy. Scale bars, 5 μm. Quantitative data are presented as mean ± SD (**P < 0.01, ***P < 0.001).

Since autophagy can promote survival under the challenge of chemotherapy (Amaravadi *et al*., 2011), we hypothesized that CD24 might regulate the response of RB cells to VCR through autophagy. To test this hypothesis, we examined the levels of autophagy in RB cells with the treatment of VCR (60 nm) for 48 h. Increased LC3‐II and decreased p62 expression were detected in control RB cells compared to RB cells with CD24 knockdown (Fig. 4A,B and Fig. S2C). To assess the effect of CD24 on the autophagic flux, we treated RB cells with CQ, a drug that prevents autophagosome–lysosome fusion and degradation. In the presence of CQ, we found increased amounts of LC3‐II and p62 in control RB cells, indicating elevated autophagic flux (Fig. 4C,D and Fig. S2D). We next evaluated autophagosomes in Y79 and WERI‐Rb‐1 cells treated with VCR (60 nm) for 48 h by transmission electron microscopy. Expectedly, an increased number of autophagosomes existed in control RB cells relative to CD24 KD RB cells (Fig. 4E,F). In addition, we established Y79 and WERI‐Rb‐1 cells that stably expressed tandem fluorescent‐tagged LC3 (RFP‐GFP‐LC3) to monitor autophagic flux. Compared with CD24 KD RB cells, control RB cells showed significantly increased numbers of autophagic puncta (Fig. 4G,H). Collectively, these evidences strongly suggested that CD24 regulates activation of autophagy in RB cells under VCR challenge.

### 3.4 CD24 blunts the sensitivity of RB to VCR via activating autophagy

**Fig. 5:**
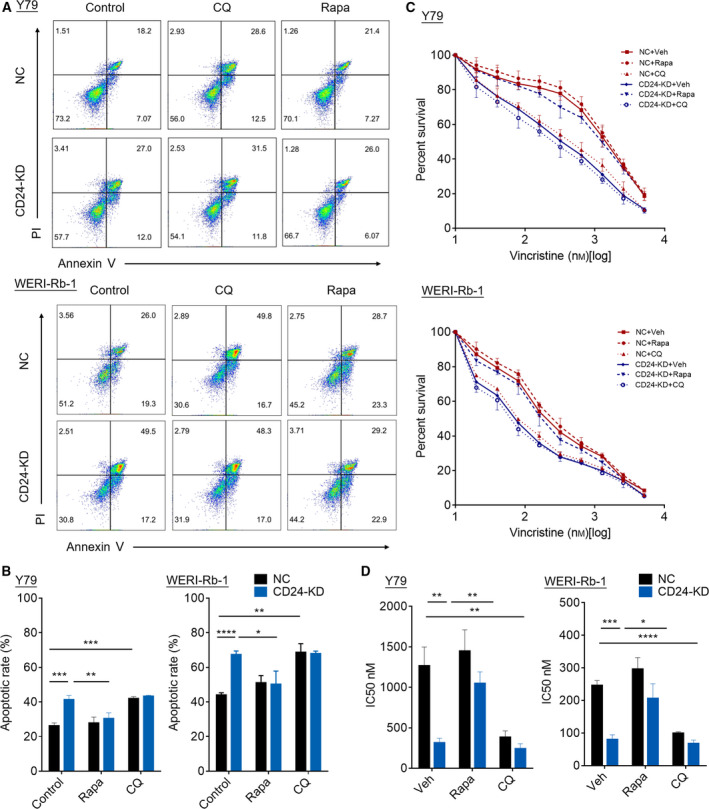
CD24 blunts the sensitivity of RB cells to VCR via activating autophagy. Cells were treated with VCR (60 nm) and rapamycin (1 μm), CQ (20 μm), or vehicle for 48 h (A, B) Apoptosis was detected by flow cytometry in Y79 cells and WERI‐Rb‐1 cells. Representative plots (A) and quantification (B) are shown (*n* = 3, Student's *t‐*test). (C) CCK‐8 assay was used to detect VCR sensitivity of cells. (D) IC50 values were determined using the CCK‐8 assay after VCR treatment (*n = *3, Student's *t*‐test). Quantitative data are presented as mean ± SD (*P < 0.05, **P < 0.01, ***P < 0.001, ****P < 0.0001).

To explore whether CD24 blunts the sensitivity of RB to VCR via autophagy pathway, we treated CD24 KD and control RB cells with CQ (an inhibitor of autophagy) or rapamycin (an inducer of autophagy acting through its inhibitory effect on mTOR) and then cultured the cells with VCR for 48 h. We found that apoptosis in CD24 KD RB cells was increased compared with control cells, and this effect was abolished by rapamycin treatment (decreased from 41.43 ± 1.96% to 30.56 ± 2.58% in Y79 cells, and decreased from 67.47 ± 1.60 to 50.33 ± 6.13% in WERI‐Rb‐1 cells). Moreover, apoptosis in control RB cells was increased under the treatment of CQ (increased from 26.38 ± 1.23% to 42.10 ± 0.82% in Y79 cells, and increased from 44.13 ± 0.98% to 68.73 ± 4.04% in WERI‐Rb‐1 cells) (Fig. 5A,B). In line with these, CCK‐8 assays showed that VCR sensitivity increased significantly in CD24 KD RB cells and control cells treated with CQ. Rapamycin blocked increased VCR sensitivity in CD24 KD RB cells (Fig. 5C,D). Therefore, CD24 impedes VCR sensitivity of RB via the autophagy pathway.

### 3.5 CD24 activates autophagy via PTEN/AKT/mTORC1 signaling pathway

**Fig. 6:**
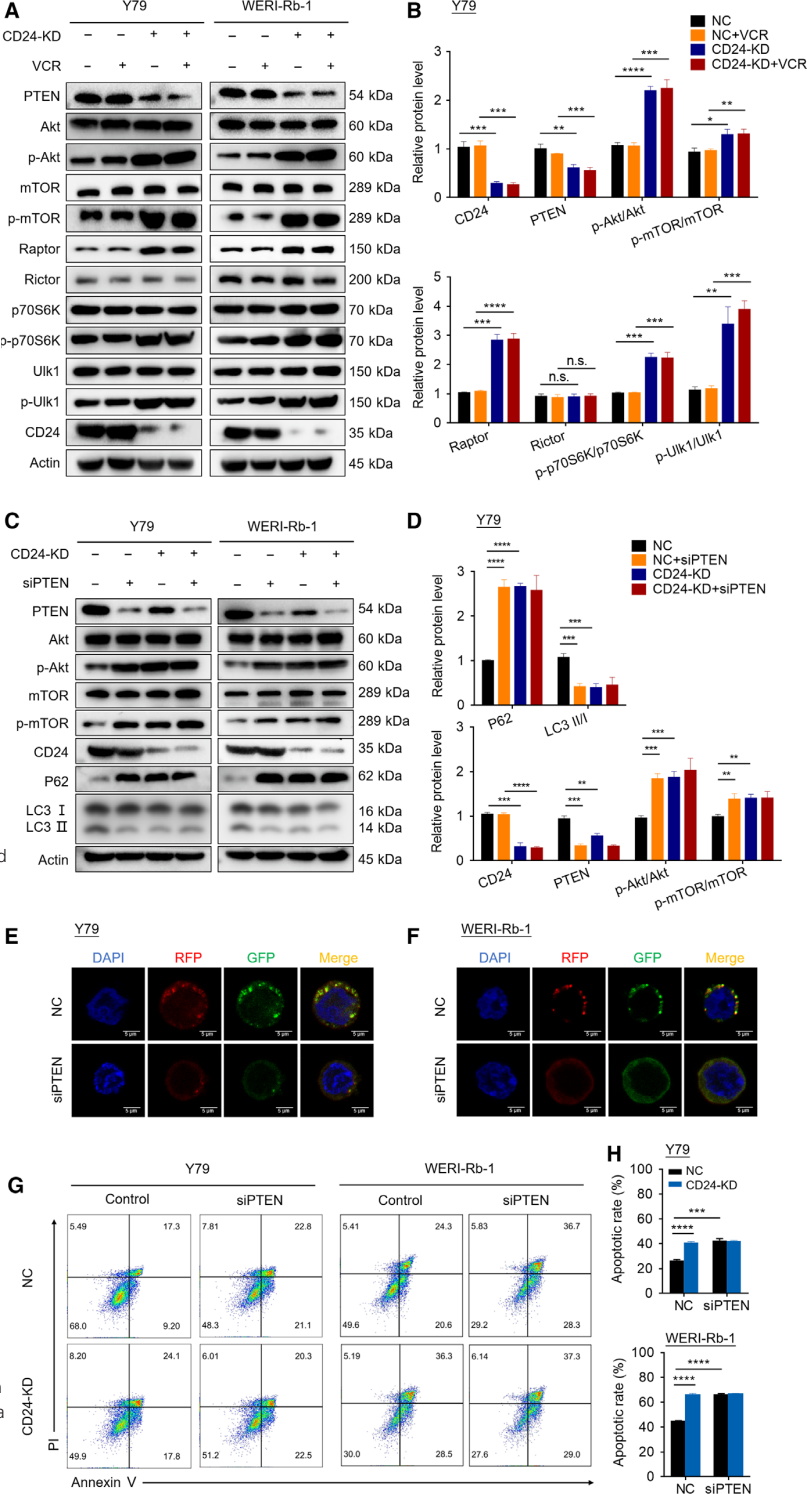
CD24 activates autophagy via PTEN/AKT/mTORC1 signaling pathway. (A) Western blotting was performed in CD24 KD and control RB cells with or without VCR (60 nm) treatment for 48 h. (B) The western blot images of Y79 cells in (A) were quantitatively analyzed using imagej software (National Institutes of Health, Bethesda, MD, USA). (C) Autophagy and target proteins were detected by western blot in CD24 KD and control RB cells. The cells were transfected with a PTEN‐targeting siRNA or control siRNA and then treated with VCR (60 nm) after 48 h. (D) The western blot images of Y79 cells in (C) were quantitatively analyzed using imagej software. (E, F) Y79 cells (E) and WERI‐Rb‐1 cells (F) that stably expressed RFP‐GFP‐LC3 fusion protein were transfected with a PTEN‐targeting siRNA or control siRNA. After incubation with VCR and treatment with CQ (20 μm) for 1 h, autophagosomes were observed under a confocal microscope in RB cells. Bar scale, 5 μm. (G, H) Apoptosis was detected by flow cytometry in CD24 KD and control RB cells. The cells were transfected with a PTEN‐targeting siRNA or control siRNA and then treated with VCR (60 nm) after 48 h. Representative plots (G) and quantification (H) are shown (*n* = 3, Student's *t‐*test). Quantitative data are presented as mean ± SD (n.s.: no significance; *P < 0.05, **P < 0.01, ***P < 0.001, ****P < 0.0001).

The PI3K‐Akt‐mTOR pathway is known to be a main regulator of autophagy (Kumar *et al*., 2015). Our RNA‐seq data indicated that differentially expressed genes (DEGs) were mainly enriched in the PI3K/AKT signaling pathway in the Environmental Information Processing category by KEGG pathway enrichment analysis (Fig. 3C). Additionally, GSEA analysis revealed that control Y79 cells tended to express less in PI3K/AKT signaling pathway, compared with CD24 KD Y79 cells (Fig. 3F). Therefore, we questioned whether CD24 might activate autophagy through downregulating PI3K‐Akt‐mTOR pathway. Since PTEN is one of the most important negative regulators of the PI3K pathway (Keyes *et al*., 2010), we examined the expression level of PTEN‐ and PI3K/AKT/mTOR‐related marker proteins in CD24 KD and control RB cells. Interestingly, CD24 knockdown not only significantly increased the expression of p‐AKT and p‐mTOR, but also downregulated PTEN expression (Fig. 6A,B and Fig. S3A).

mTOR forms two complexes called mTORC1 (mTOR complexed with Raptor) and mTORC2 (mTOR complexed with Rictor) (Kaibori *et al*., 2015). A large body of work demonstrates that the inhibition of mTOR signaling plays an essential role in autophagy induction, as mTORC1 acts as a central regulator in autophagy induction (Yang *et al*., 2011). We next monitored mTORC1 and mTORC2 activity via the protein levels of mTOR complex subunits Raptor and Rictor. In both cell types, we observed that the protein level of Raptor was increased by CD24 knockdown, whereas that of Rictor was not affected. Raptor binds to mTOR substrates, such as 4E‐BP1 and p70 S6 kinase (p70S6K), through their TOR signaling (TOS) motifs and is required for mTOR‐mediated substrate phosphorylation (Beugnet *et al*., 2003; Kim *et al*., 2002). In addition, suppressing the activation of P70S6K, a key downstream kinase of mTOR, can promote autophagy (Sun *et al*., 2018). We detected the expression of p70S6K and p‐p70S6k. The results showed that the p‐P70S6K/P70S6K ratios were increased by CD24 knockdown. It has been reported that AMPK and mTORC1 regulate autophagy through coordinated phosphorylation of Ulk1. Ulk1 is a key regulator in autophagy initiation. AMPK promotes autophagy by directly activating Ulk1 through phosphorylation of Ser 317 and Ser 777. High mTORC1 activity prevents Ulk1 activation by phosphorylating Ulk1 Ser 757 and disrupting the interaction between Ulk1 and AMPK, and eventually leads to autophagy inhibition (Kim *et al*., 2011). Next, we determined the effect of CD24 knockdown on Ulk1 Ser 757 phosphorylation. We found that CD24 knockdown induced a robust Ulk1 phosphorylation at Ser 757 (Fig. 6A,B and Fig. S3A).

To investigate the role of PTEN in the CD24‐induced autophagy process, siRNA for the *PTEN* gene was used to knock down PTEN protein expression. Western blotting revealed that silencing PTEN significantly activated the AKT/mTOR pathway, suppressed CD24‐induced conversion of LC3‐I to LC3‐II, and simultaneously increased p62 protein expression in RB cells treated with VCR (Fig. 6C,D and Fig. S3B). Moreover, silencing PTEN reduced the accumulation of autophagosomes in Y79 cells (Fig. 6E) and WERI‐Rb‐1 cells (Fig. 6F) under VCR challenge, compared to controls. Additionally, it increased VCR‐induced apoptosis in RB cells (Fig. 6G,H).

Together, these data indicated that CD24 activates autophagy via PTEN/AKT/mTORC1 signaling pathway, and eventually decreased the sensitivity of RB cells to VCR.

### 3.6 CD24 recruits PTEN to the lipid raft domain

**Fig. 7:**
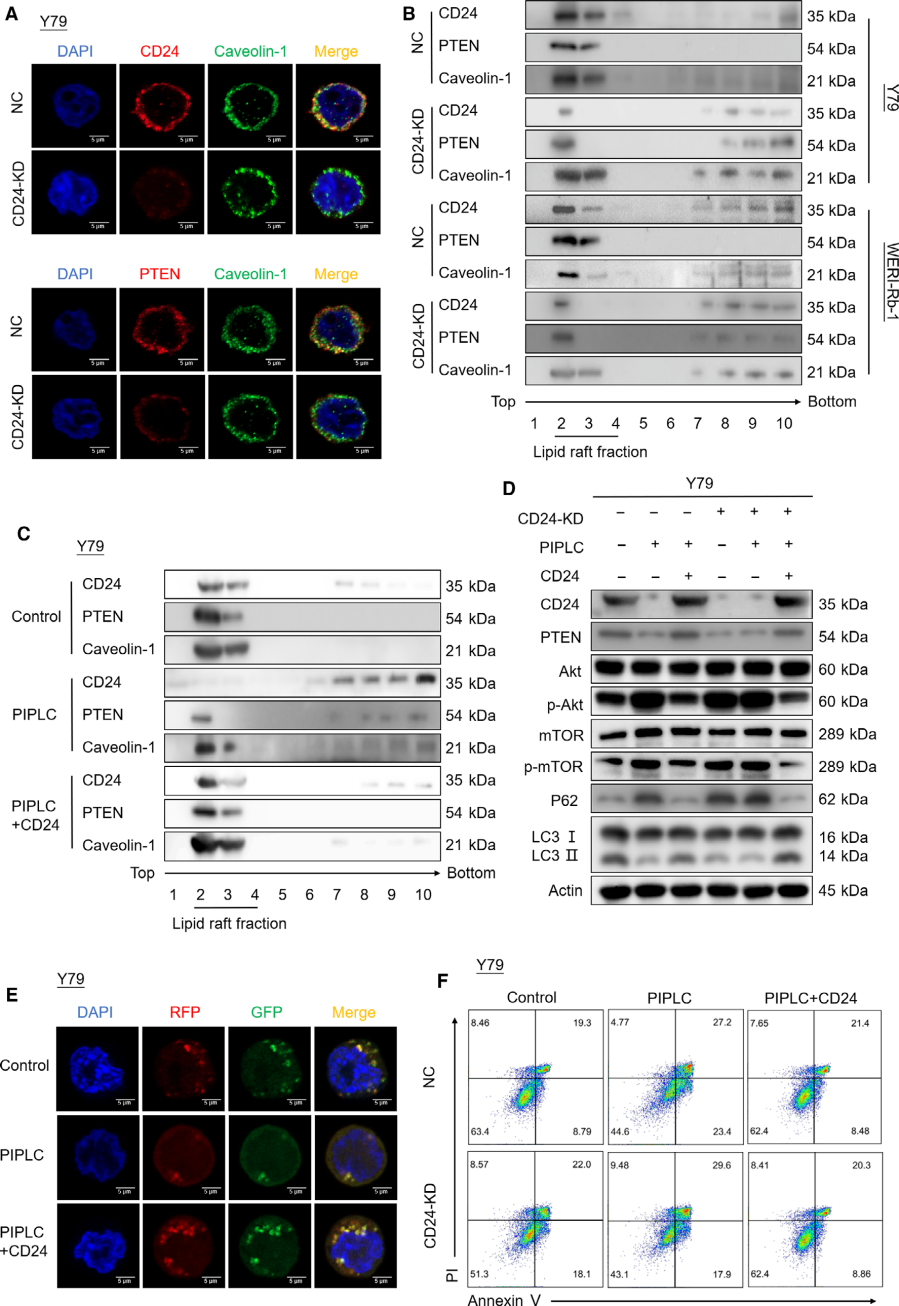
CD24 recruits PTEN to the lipid raft domain, and its function depends on GPI anchorage to lipid rafts. (A) Immunofluorescent staining of CD24 KD and negative control (NC) Y79 cells. Scale bars, 5 μm. (B) Lipid raft fractions were analyzed by western blotting using antibodies against CD24, PTEN, and caveolin‐1. (C) Lipid raft fractions were subjected to western blotting. Y79 cells were incubated with PIPLC (4 U·mL^−1^) for 60 min at 37 °C and then transduced with CD24 overexpression plasmid. (D) PTEN, Akt/p‐Akt, mTOR/p‐mTOR, and autophagy proteins were analyzed by western blotting. CD24 KD and negative control (NC) Y79 cells were treated with PIPLC and then transduced with CD24 overexpression plasmid. (E) Y79 cells were treated with PIPLC and then transduced with CD24 overexpression plasmid. After incubation with VCR and treatment with CQ (20 μm) for 1 h, autophagosomes were observed by transmission electron microscopy. Scale bars, 0.5 μm. (F) Apoptosis was detected by flow cytometry in CD24 KD and control Y79 cells. The cells were treated with PIPLC, transduced with CD24 overexpression plasmid, and then treated with VCR (60 nm) for 48 h.

Glycosylphosphatidylinositol‐anchored proteins, including CD24, are localized to lipid rafts and play a central role in signal transduction pathways (Suzuki, 2015). PTEN is a lipid phosphatase that dephosphorylates phosphatidylinositol 3,4,5‐trisphosphate (PIP3) to PI (4,5) P2 and antagonizes the PI3K‐AKT pathway. Its lipid phosphatase activity is associated with plasma membrane localization (Rahdar *et al*., 2009). It has been reported that ceramide can stimulate PTEN to transfer to lipid rafts in the plasma membrane, where it can act to reduce the levels of PIP3 necessary for the activation of Akt (Goswami *et al*., 2005). We hypothesized that CD24 may downregulate the PI3K‐Akt‐mTOR pathway by recruiting PTEN to the lipid raft domain. To test this hypothesis, we employed immunofluorescence staining to demonstrate the colocalization of CD24, PTEN, and caveolin‐1, a marker of lipid rafts. We found that both CD24 and PTEN were localized to lipid rafts in RB cells, whereas the colocalization of PTEN and caveolin‐1 was diminished in CD24 KD RB cells (Fig. 7A and Fig. S4A). In addition, we fractionated lipid rafts by a sucrose gradient assay. Western blot analysis of the gradient fractions with an anti‐caveolin‐1 antibody showed that lipid rafts excluding other components were distributed in 15–20% sucrose fractions (Fig. 7B). Consistent with the above result, both CD24 and PTEN could be detected in lipid rafts. Furthermore, CD24 knockdown significantly reduced enrichment of PTEN in lipid rafts. Collectively, these results suggested that CD24 recruits PTEN to the lipid raft domain.

### 3.7 CD24 function depends on GPI anchorage to lipid rafts

We next sought to explore whether GPI anchor was necessary for CD24 function. CD24 KD and control RB cells were incubated with phosphatidylinositol‐specific phospholipase C (PIPLC), an enzyme that cleaves GPI‐anchored proteins from GPI in the cell membrane, and then transfected with empty vector control or CD24 overexpression plasmid. Western blotting revealed that CD24 was released from lipid rafts after PIPLC treatment, resulting in less enrichment of PTEN in lipid rafts. This effect was abolished by CD24 overexpression plasmid transfection in Y79 cells (Fig. 7C) and WERI‐Rb‐1 cells (Fig. S4B). Moreover, PIPLC treatment significantly suppressed autophagy via the PTEN/AKT/mTOR signaling pathway and increased apoptosis in RB cells under VCR chemotherapy. By transfecting PIPLC‐treated RB cells with CD24 overexpression plasmid, autophagy was activated and apoptosis was reduced in RB cells cultured with VCR (Fig. 7D–F and Fig. S4C–F). Thus, these data supported that CD24 function depends on its anchorage to lipid rafts.

### 3.8 CD24 impairs the sensitivity to VCR in RB *in vivo*

**Fig. 8:**
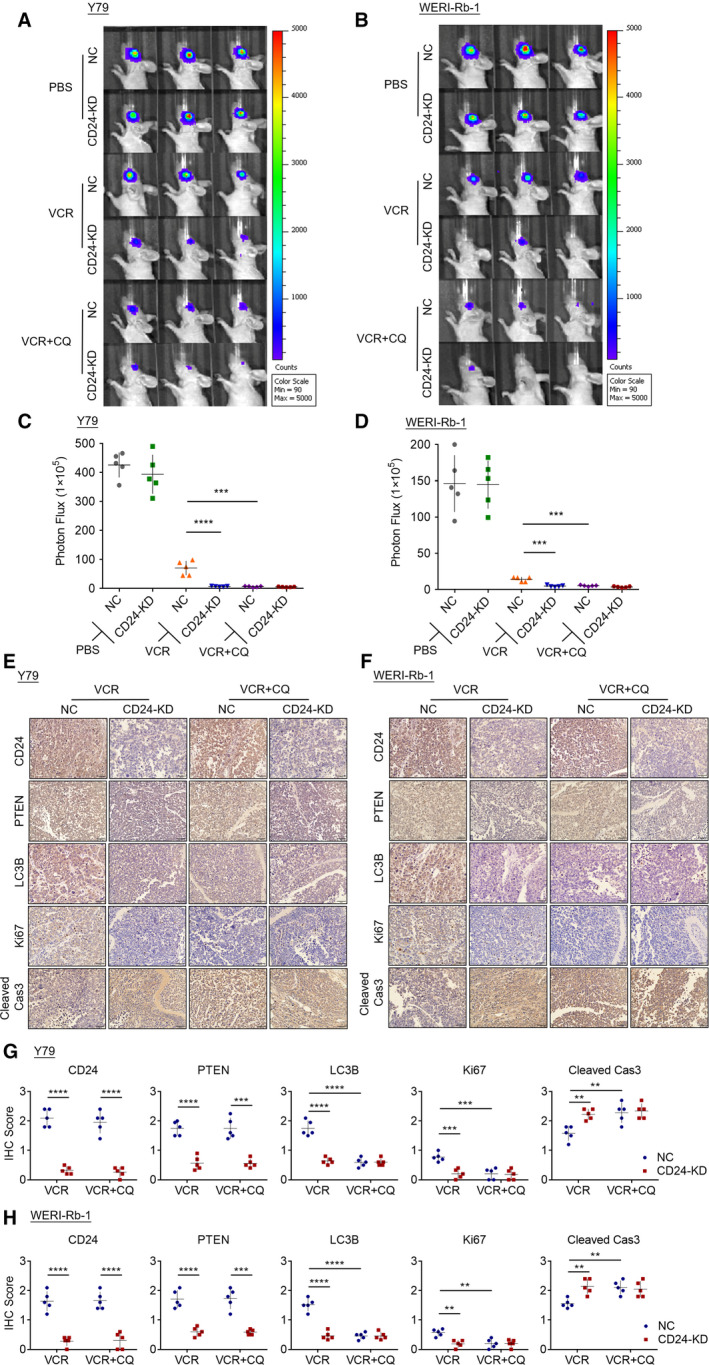
CD24 impairs the sensitivity of RB cells to VCR *in vivo*. CD24 KD and control RB cells were injected into the vitreous of BALB/c mice, and intraperitoneal injection of PBS or VCR (1 mg·kg^−1^) + CQ (30 mg·kg^−1^) was administrated twice per week for 3 weeks. Tumor burden was measured by bioluminescent imaging (*n* = 5, Student's *t‐*test). (A, B) Representative bioluminescent images of RB tumors in mice under different conditions. (C, D) Statistical analysis of tumor burden in different groups (*n* = 5, Student's *t*‐test). (E, F) Representative IHC staining for CD24, PTEN, LC3B, Ki67, and cleaved caspase‐3 expression in xenografts. Scale bars, 50 μm. (G, H) Quantitative analysis of CD24, PTEN, LC3B, Ki67, and cleaved caspase‐3 protein expression based on staining index in xenografts (*n* = 5, Student's *t*‐test). Quantitative data are presented as mean ± SD (*P < 0.05, **P < 0.01, ***P < 0.001, ****P < 0.0001).

To study the role of CD24 in regulating autophagy activation and VCR sensitivity of RB cells *in vivo*, we inoculated CD24 KD and control RB cells into the vitreous bodies of nude mice and intraperitoneally injected with VCR or CQ. Very impressively, the xenografts derived from CD24 KD RB cells manifested a dramatically increased sensitivity to VCR compared with the control group (Fig. 8A–D). In addition, CD24‐induced diminished response to VCR therapy was rescued by CQ treatment. Similarly, we detected a significantly increased expression of ki67 and a significantly decreased expression of cleaved caspase‐3 in control group cells compared with other groups under VCR therapy (Fig. 8E–H). Thus, CD24 affects the sensitivity of RB cells in response to VCR therapy through autophagy *in vivo*. We further detected the protein expression of CD24, PTEN, and cleaved LC3 in the tumor tissues by IHC. The expression of PTEN and cleaved LC3 was decreased after CD24 knockdown (Fig. 8E–H). The results clearly suggested that CD24 induces autophagy by regulating PTEN.

## 4 Discussion

Due to its heat resistance, CD24 was first identified as a heat‐stable antigen in 1978 (Springer *et al*., 1978). The mature human CD24 protein has 32 residues and is localized to the plasma membrane via a GPI anchor (Kay *et al*., 1991). CD24 is expressed on hematopoietic cells, immature neuronal cells, and numerous types of cancer cells (Fang *et al*., 2010). Functionally, it is considered to play a critical role in regulating lymphocyte development, modulating neurite outgrowth, and altering cancer cell interactions (Gilliam *et al*., 2017). Recently, several studies have shown enhanced CD24 expression in various cancers including RB, where CD24 expression is associated with severity of disease (Bektas *et al*., 2010; Liu *et al*., 2011; Riener *et al*., 2010). It has been reported that CD24 is highly expressed and interacts with the inhibitory receptor sialic acid‐binding Ig‐like lectin 10 (Siglec‐10), which is expressed by tumor‐associated macrophages to promote immune evasion in ovarian cancer and breast cancer (Barkal *et al*., 2019). Previous study has found that there is no CD24 expression in non‐neoplastic retina, and the level of CD24 is significantly increased in RB tissues with high risk of extraocular relapse compared to those with low risk of extraocular relapse (Li *et al*., 2012). In our study, we found that CD24 is overexpressed in RB and may be involved in the pathogenesis of RB. These data reveal that CD24 can be a promising target for cancer therapy.

Accumulating evidence has found an association between CD24 and cancer chemosensitivity. In HCC, CD24 is upregulated in chemoresistant xenograft tumors and CD24^+^ HCC cells possess properties characteristic of stem cells (Lee *et al*., 2011). In endometrial cancer cell lines, CD24 promotes the expression of ATP‐binding cassette (ABC) transporters via the Met signaling cascade, ultimately leading to cancer drug resistance (Ono *et al*., 2015). In MCF‐7 breast cancer cells, CD24 inhibition reduces chemosensitivity to 5‐fluorouracil, and ABC transporter expression appears not to contribute to this mechanism (Onishi *et al*., 2017). In TNBC, CD24 affects chemosensitivity according to drug type and may regulate the drug sensitivity of tumor cells, in part through modulation of cellular autophagy status (Deng *et al*., 2017).

Chemotherapy is the standard treatment for RB. However, the presently available chemotherapeutics, such as vincristine (VCR), etoposide, and carboplatin, often result in drug resistance (Murphree *et al*., 1996). Therefore, exploration of the mechanisms underlying chemoresistance may significantly improve the survival rate and prognosis of the patients. In the present study, we investigated whether CD24 is associated with the sensitivity of RB cells to VCR. It was observed that CD24 knockdown significantly enhanced the inhibitory effect of VCR on Y79 and WERI‐Rb‐1 cells.

Recent studies have demonstrated that autophagy can be activated as a protective mechanism to mediate chemotherapy resistance in cancer cells (Sui *et al*., 2013). The autophagy process is also activated in the chemoresistant RBs. In our study, CD24 knockdown dramatically decreased autophagic flux and inhibited the autophagosome formation in RB cells under VCR challenge. The sensitivity of RB cells to VCR can be restored by autophagy inhibitors, which strongly indicates that the autophagy process plays a critical role in RB cell sensitivity to VCR therapy. We further explored mechanisms how CD24 regulates autophagy process. Various signaling pathways have been implicated in the regulation of autophagy (Kumar *et al*., 2015). The PI3K‐Akt pathway and its downstream effector, the mTOR complex, have emerged as the main inhibitory regulators of autophagy (Wu *et al*., 2009). PTEN, a classical tumor suppressor protein, has been found to positively regulate autophagy by inhibiting the PI3K‐Akt pathway (Arico *et al*., 2001). Multiple studies have confirmed the role of PTEN in drug resistance (Li *et al*., 2017b; Shen *et al*., 2017; Xu *et al*., 2017). We investigated whether CD24 enhanced autophagy via the PTEN/PI3K/Akt pathway in RB cells. We showed that downregulation of CD24 inhibited the abundance of PTEN and elevated the phosphorylation levels of PTEN, AKT, and mTOR. PTEN depletion obviously inhibited autophagy and sensitized RB cells to VCR therapy. Raptor may have the regulatory role in the PI3K/Akt/mTORC1 pathway. It has been reported that fisetin suppresses PI3K/Akt/mTOR signaling in human non‐small‐cell lung cancer cells. The protein expression of Raptor was decreased by 50–97% on fisetin treatment (Khan *et al*., 2012). Notoginsenoside R7 treatment increased PTEN activation and downregulated mTOR phosphorylation and decreased Raptor expression in HeLa cells (Li *et al*., 2017a). Aspirin can regulate the proliferation, apoptosis, and autophagy of colorectal cancer (CRC) cells through the PI3K/Akt/Raptor pathway, affecting PIK3CA‐mutant CRC. As the concentration of aspirin increases, the protein expression of Raptor decreases (Chen *et al*., 2020). This change in Raptor expression may be at the transcriptional level. The Raptor promoter contains multiple transcription factor motifs, and Raptor expression is reportedly regulated via miRNA binding to the 3‐UTR of Raptor mRNA in erythroid cells (Bianchi *et al*., 2015). Our results also showed that CD24 knockdown upregulated Raptor protein expression level and increased mTORC1 activation.

Since PTEN regulates the PI3K/Akt pathway through lipid phosphatase activity, it may be required to localize to the plasma membrane at the appropriate site for access to lipid substrates and regulate Akt. It has been reported that PTEN function can be influenced by modifying its lipid membrane binding (Milella *et al*., 2015). It was demonstrated that PTEN binds to MAGI‐2 through an interaction between the C terminus of PTEN and the second PDZ domain of MAGI‐2, which contributes to the recruitment and maintenance of membrane association of PTEN, and ultimately enhances the ability of PTEN to suppress Akt activation (Wu *et al*., 2000). MEK1/MAGI‐1/PTEN complex mediates the translocation of PTEN to the membrane and thereby controls PIP3 accumulation and AKT signaling (Zmajkovicova *et al*., 2013). Due to the absence of cytoplasmic domain, CD24 was considered to exert its functions by regulating the association of different proteins with lipid rafts that act as signaling platforms. It has been reported that CD24 acts as a ‘gate‐keeper’ for lipid raft domains (Runz *et al*., 2008). CD24 regulates the activity of the chemokine receptor CXCR4 by affecting its localization in the plasma membrane lipid rafts of pre‐B cells and breast carcinoma cells (Schabath *et al*., 2006). It also recruits β1 integrin into lipid raft domains to augment β1‐dependent cell motility and stimulate transmigration and invasion across a monolayer of endothelial cells. Similarly, CD24 recruits p‐Met to the lipid raft domain, resulting in the drug resistance of endometrial cancer (Ono *et al*., 2015). We herein demonstrated for the first time that CD24 was responsible for the recruitment of PTEN to the lipid raft domain and its function depends on GPI anchorage to lipid rafts. However, the mechanisms by which CD24 recruits PTEN to the lipid raft domain remain to be further studied.

## 5 Conclusions

In summary, CD24 is more highly expressed in RB cells and tissues and is associated with response of RB cells to VCR‐based chemotherapy. Suppression of CD24 expression eliminated autophagy via the PTEN/Akt/mTORC1 pathway and significantly increased the sensitivity of RB cells to VCR. Our findings suggest that CD24 is an important regulator of autophagy and may be a novel target for more effective RB chemotherapy.

## Conflict of interest

The authors declare no conflict of interest.

## Author contributions

JS performed experiments, analyzed data, and wrote the paper. HX contributed to the sample collection and interpretation of the data. DF helped with the experiments. QL, YC, and QS initiated the study, designed experiments, and wrote the paper. JL and ZZ revised the manuscript. All authors read and approved the final manuscript.

## Ethics approval and consent to participate

All experiments were performed in accordance with a protocol approved by the Institutional Animal Care and Use Committee of Nanjing Medical University.

## Supporting information


**Fig. S1.** CD24 impedes the sensitivity of RB cells to VCR.
**Fig. S2.** CD24 regulates autophagy activation in RB cells under VCR challenge
**Fig. S3.** CD24 activates autophagy via PTEN/AKT/mTORC1 signaling pathway.
**Fig. S4.** CD24 recruits PTEN to the lipid raft domain, and its function depends on GPI‐anchorage to lipid rafts.
**Table S1.** Antibodies or reagents.
**Table S2.** Characteristics of Tissue Microarray Specimens.
**Table S3.** Primers used for quantitative real‐time PCR.
**Table S4.** Sequences of siRNAs and shRNAs.
