# Supplementary material for: CD24 blunts the sensitivity of retinoblastoma to vincristine by modulating autophagy
Source: Mol Oncol. 2020 Jun 13;14(8):1740–59. doi: 10.1002/1878-0261.12708 (PMC7400807; doi:10.1002/1878-0261.12708)
Supplement: Supplementary file 1 — Fig. S1. CD24 impedes the sensitivity of RB cells to VCR. Fig. S2. CD24 regulates autophagy activation in RB cells under VCR challenge Fig. S3. CD24 activates autophagy via PTEN/AKT/mTORC1 signaling pathway. Fig. S4. CD24 recruits PTEN to the lipid raft domain, and its function depends on GPI‐anchorage to lipid rafts. Table S1. Antibodies or reagents. Table S2. Characteristics of Tissue Microarray Specimens. Table S3. Primers used for quantitative real‐time PCR. Table S4. Sequences of siRNAs and shRNAs. [file MOL2-14-1740-s001.pdf]

Supporting information

Supplementary Tables

Table S1. Antibodies or reagents.

Table S2. Characteristics of Tissue Microarray Specimens.

Table S3. Primers used for quantitative real-time PCR.

Table S4. Sequences of siRNAs and shRNAs.

Supplementary Figures and Figure Legends

Fig. S1. CD24 impedes the sensitivity of RB cells to VCR.

Fig. S2. CD24 regulates autophagy activation in RB cells under VCR challenge

Fig. S3. CD24 activates autophagy via PTEN/AKT/mTORC1 signaling pathway.

Fig. S4. CD24 recruits PTEN to the lipid raft domain, and its function depends on GPI-anchorage to lipid rafts.

Table S1

Antibodies or reagents

Antibodies or reagents	Source	Catalog number
Anti-CD24	Santa Cruz Biotechnology	sc-11406
Anti-CD24	Invitrogen	12-0247-41
Anti-Actin	Cell Signaling Technology	4970
Anti-Cleaved caspase-3	Cell Signaling Technology	9664
Anti-Cleaved PARP	Cell Signaling Technology	5625
Anti-Tubulin	Proteintech	66031-1-lg
Anti-P62	Abcam	ab109012
Anti-LC3B	Abcam	ab48394
Anti-Beclin1	Cell Signaling Technology	3495
Anti-Atg3	Cell Signaling Technology	3415
Anti-Atg5	Cell Signaling Technology	12994
Anti-Atg12	Cell Signaling Technology	4180
Anti-Atg16L1	Cell Signaling Technology	8089
Anti-Caveolin-1	Cell Signaling Technology	3267
Anti-PTEN	Cell Signaling Technology	9188
Anti-AKT	Cell Signaling Technology	4691
Anti-p-AKT (Thr308)	Cell Signaling Technology	13038
Anti-mTOR	Cell Signaling Technology	2983
Anti-p-mTOR (Ser2448)	Cell Signaling Technology	5536

Anti-Raptor	Cell Signaling Technology	2280
Anti-Rictor	Cell Signaling Technology	2114
Anti-p70S6K	Cell Signaling Technology	2708
Anti-p-p70S6K	Cell Signaling Technology	9206
Anti-ULK1	Cell Signaling Technology	6439
Anti-p-ULK1	Cell Signaling Technology	6888
Vincristine	Selleck Chemicals	S1241
Chloroquine	Selleck Chemicals	S4157
Rapamycin	Selleck Chemicals	S1039
PIPLC	Sigma-Aldrich	P5542

Table S2

Characteristics of Tissue Microarray Specimens

Sample No.	Age	Sex	Pathology diagnosis	TNM	Stage
1	15	F	Retinoblastoma	T3cN0M0	IIC
2	60	M	Retinoblastoma	T4bN0M0	IIIB
3	2	F	Retinoblastoma	T2N0M0	IB
4	11	F	Retinoblastoma	T2N0M0	IB
5	28	M	Retinoblastoma	T3cN0M0	IIC
6	3	M	Retinoblastoma	T2N0M0	IB
7	2	M	Retinoblastoma	T3aN0M0	IIA
8	3	M	Retinoblastoma	T2N1M0	IV
9	27	M	Retinoblastoma	T3N0M0	II
10	2	M	Retinoblastoma	T2N0M0	IB
11	74	M	Retinoblastoma	T2N0M0	IB
12	70	M	Retinoblastoma	T3bN0M0	IIB
13	8 Mon.	M	Retinoblastoma with necrosis	T3aN0M0	IIA
14	4	M	Retinoblastoma	TIN0M0	IA
15	63	M	Normal retina tissue		
16	44	M	Normal retina tissue		
17	1	M	Normal cornea tissue		
18	62	M	Normal eyeball wall tissue		
19	2	F	Normal eyeball wall tissue		

20	2	F	Normal eyeball wall tissue

Table S3

Primers used for quantitative real-time PCR

Target Genes	Forward Primer (5’-3’)	Reverse Primer (5’-3’)
β-actin	ATTGGCAATGAGCGGTTC	GGATGCCACAGGACTCCAT
CD24	TGAAGAACATGTGAGAGGTTTGAC	GAAAACTGAATCTCCATTCCACAA
Beclin1	CCATGCAGGTGAGCTTCGT	GAATCTGCGAGAGACACCATC
Atg3	GACCCCGGTCCTCAAGGAA	TGTAGCCCATTGCCATGTTGG
Atg5	AAAGATGTGCTTCGAGATGTGT	CACTTTGTCAGTTACCAACGTCA
Atg12	CTGCTGGCGACACCAAGAAA	CGTGTTCGCTCTACTGCCC
Atg16L	AACGCTGTGCAGTTCAGTCC	AGCTGCTAAGAGGTAAGATCCA

Table S4

Sequences of siRNAs and shRNAs

Name	Sequence (5’-3’)
siCD24-1	ACAACTGGAACTTCAAGTAAC
siCD24-2, shCD24	GGGCAAUGAUGAAUGAGAATT
siPTEN	CGCCAAAUUUAAUUGCAGATT

Supplementary figures and figure legends

Fig. S1

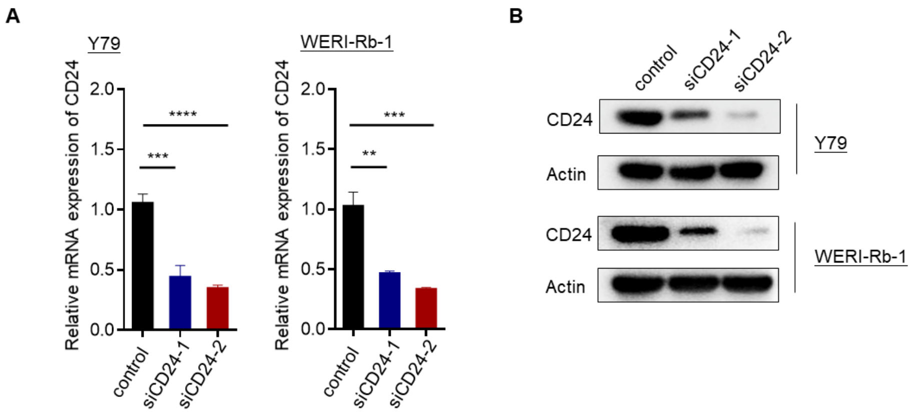

Figure S1. CD24 impedes the sensitivity of RB cells to VCR, Related to Figure 2. (A, B) Stable inhibitory efficiency of siRNAs against CD24 in Y79 and WERI-Rb-1 cells was detected by qPCR (*n*=3, Student's *t*-test) and western blotting. Quantitative data are presented as mean ± SD (*** P <0.001, **** P <0.0001).

Fig. S2

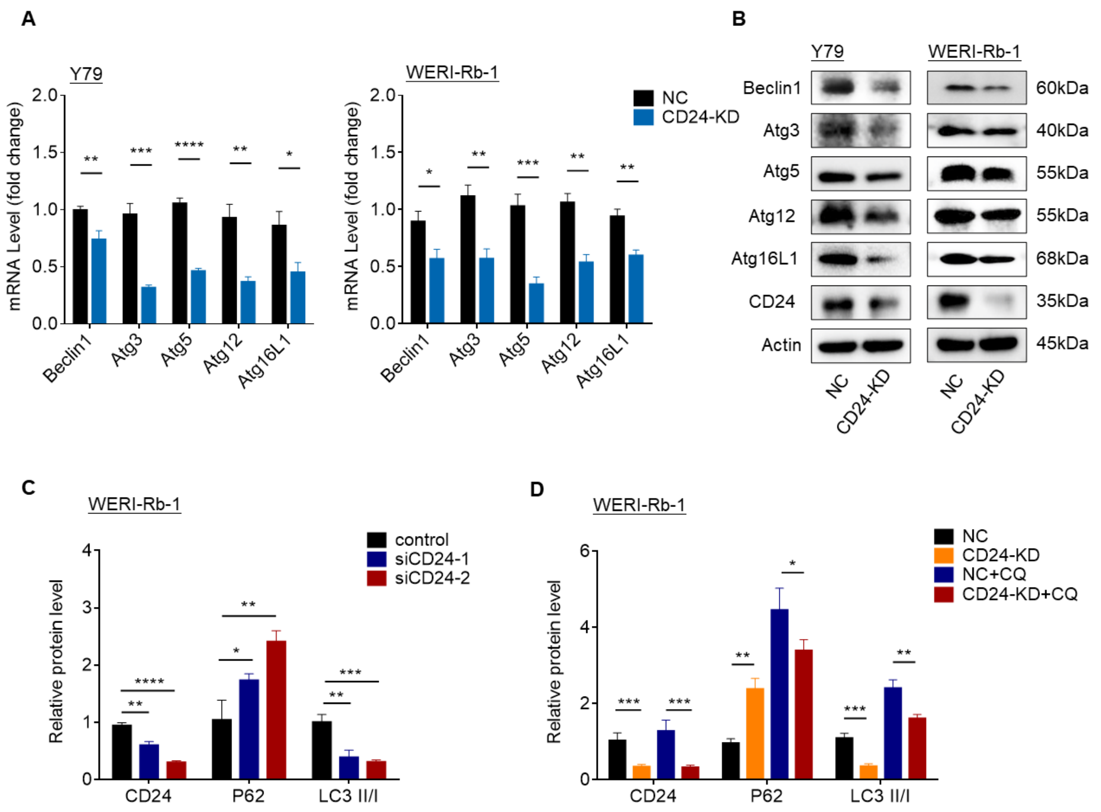

Figure S2. CD24 regulates autophagy activation in RB cells under VCR challenge, Related to Figure 4. (A, B) The expression levels of autophagy-related protein in CD24 KD and control RB cells were detected by qPCR (A) (n=3, Student's t-test) and western blotting (B). (C) The Western blot images of WERI-Rb-1 cells in Figure 4A were quantitatively analyzed using ImageJ software. (D) The Western blot images of WERI-Rb-1 cells in Figure 4C were quantitatively analyzed using ImageJ software. Quantitative data are presented as mean ± SD (* $P < 0.05$, ** $P < 0.01$, *** $P < 0.001$, **** $P < 0.0001$)

Fig. S3

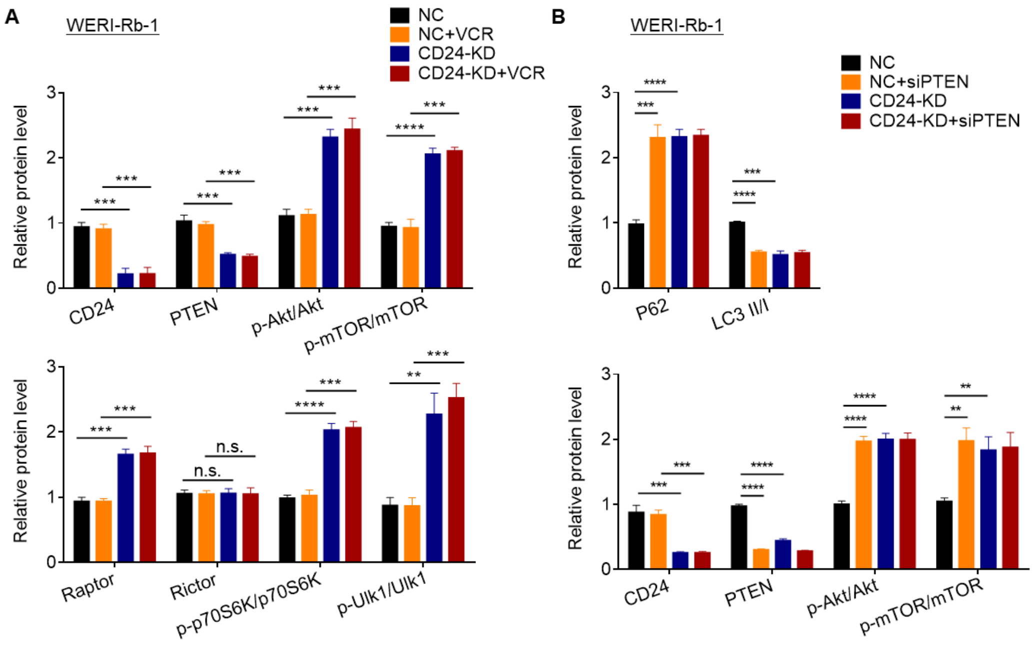

Figure S3. CD24 activates autophagy via PTEN/AKT/mTORC1 signaling pathway, Related to Figure 6. (A) The Western blot images of WERI-Rb-1 cells in Figure 6A were quantitatively analyzed using ImageJ software. (B) The Western blot images of WERI-Rb-1 cells in Figure 6C were quantitatively analyzed using ImageJ software. Quantitative data are presented as mean ± SD (n.s. no significance, * $P < 0.05$, ** $P < 0.01$, *** $P < 0.001$, **** $P < 0.0001$)

Fig. S4

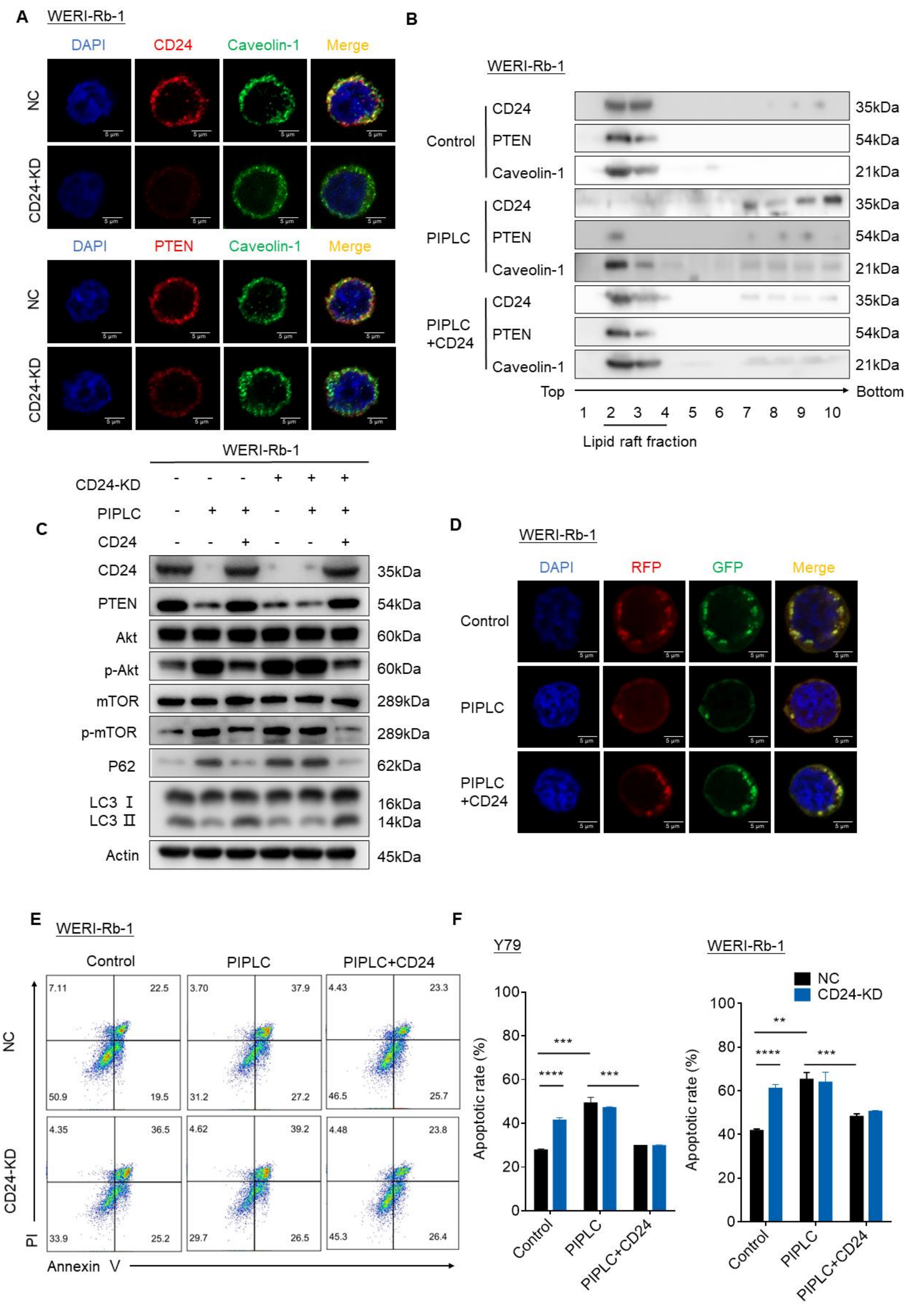

Figure S4. CD24 recruits PTEN to the lipid raft domain, and its function depends on GPI-anchorage to lipid rafts, Related to Figure 7. (A) Immunofluorescent

staining of CD24 KD and negative control (NC) WERI-Rb-1 cells. Scale bars, 5 μm. (B) Lipid raft fractions were subjected to western blotting. WERI-Rb-1 cells were treated with PIPLC (4U/mL) for 60 min at 37°C and then transduced with CD24 overexpression plasmid. (C) PTEN, Akt/p-Akt, mTOR/p-mTOR and autophagy proteins were analyzed by western blotting. CD24 KD and negative control (NC) WERI-Rb-1 cells were treated with PIPLC and then transduced with CD24 overexpression plasmid. (D) WERI-Rb-1 cells were treated with PIPLC and then transduced with CD24 overexpression plasmid. After incubation with VCR and treatment with CQ(20 μM) for 1 h, autophagosomes were observed by transmission electron microscopy. Scale bars, 0.5 μm. (E, F) Apoptosis was detected by flow cytometry in CD24 KD and control RB cells. The cells were treated with PIPLC, transduced with CD24 overexpression plasmid, and then treated with VCR (60 nM) after 48 h. Representative plots of WERI-Rb-1 cells (E) and quantification (F) are shown (n=3, Student's t-test). Quantitative data are presented as mean ± SD (** $P < 0.01$, *** $P < 0.001$, **** $P < 0.0001$).
